# Noncytolytic CD8^+^ Cell Mediated Antiviral Response Represents a Strong Element in the Immune Response of Simian Immunodeficiency Virus-Infected Long-Term Non-Progressing Rhesus Macaques

**DOI:** 10.1371/journal.pone.0142086

**Published:** 2015-11-09

**Authors:** Aneela Javed, Nicole Leuchte, Berit Neumann, Sieghart Sopper, Ulrike Sauermann

**Affiliations:** 1 Atta-ur-Rahman school of Applied Biosciences(ASAB), National University of Science and Technology (NUST), Islamabad, Pakistan; 2 Deutsches Primatenzentrum GmbH, Leibniz-Institut für Primatenforschung, Abteilung Infektionsmodelle, Göttingen, Germany; 3 Tumor Immunology Lab, Hematology and Oncology, Medical University Innsbruck and Tyrolean Cancer Research Institute, Innsbruck, Austria; CEA, FRANCE

## Abstract

The ability of long term non progressors to maintain very low levels of HIV/SIV and a healthy state, involves various host genetic and immunological factors. CD8+ non-cytolytic antiviral response (CNAR) most likely plays an important role in this regard. In order to gain a deeper insight into this unique phenomenon, the ability of CD8+ T cells to suppress viral replication *in vitro* was investigated in 16 uninfected, longitudinally in 23 SIV-infected long-term non-progressing (LTNPs), and 10 SIV-infected rhesus macaques with progressing disease. An acute infection assay utilizing CD4^+^ cells from MHC-mismatched monkeys to avoid cytolytic responses was employed. The study has identified CNAR as a long-term stable activity that inversely correlated with plasma viral load. The activity was also detected in CD8^+^ cells of uninfected macaques, which indicates that CNAR is not necessarily a virus specific response but increases after SIV-infection. Physical contact between CD4^+^ and CD8^+^ cells was mainly involved in mediating viral inhibition. Loss of this activity appeared to be due to a loss of CNAR-expressing CD8+ cells as well as a reduction of CNAR-responsive CD4+ cells. In contrast, *in vitro* viral replication did not differ in CD4+ cells from un-infected macaques, CNAR(+) and CNAR(-) LTNPs. A role for transitional memory cells in supporting CNAR in the macaque model of AIDS was questionable. CNAR appears to represent an important part of the immune response displayed by CD8+ T cells which might be underestimated up to now.

## Introduction

Following infection with human (HIV) or simian immunodeficiency virus (SIV), the rate of clinical disease progression varies between individuals. A small subset of HIV/SIV infected individuals termed elite controllers [1, 2], elite suppressors [3] and long term non progressors (LTNPs; [4]) can efficiently control viral replication and do not show any clinical symptoms of immunodeficiency for prolonged periods. The mechanisms underlying this unique suppression of viral replication are not completely elucidated and are certainly multifactorial. Identification of these factors will inform the development of preventive or therapeutic vaccines.

CD8^+^ T cells play a critical role in controlling viral replication. Classical studies indicated that CD8^+^ cells from infected individuals can lyse autologous target cells and play therefore an important role in limiting viral replication [5, 6]. Numerous studies revealed that certain *MHC* class I alleles which are responsible for initiating and controlling a CTL-response, represent the strongest host factor associated with control of viral replication (e.g.[7]). The immunological pressure exerted on the virus was further demonstrated by detection of sequence variations resulting in an escape from MHC-mediated immune control [8–11]. Vaccine experiments in nonhuman primates further demonstrated that CTL-responses can hinder acquisition of pathogenic SIV [12]. Moreover, the quality of the immune response such as the presence of polyfunctional CD8^+^ T cells adds to superior containment of the virus [13–16]. Analysis of phenotypic and functional properties of CD8^+^ T cell subsets showed that central (CD28^high^ CD95^+^) and effector memory cells (CD28^low/-^ CD95^+^), and possibly transitional memory cells (CD27^+^ CD28^−^ CD45RA^−^ CCR7^−^) can significantly inhibit viral replication *in vitro* [17–20].

Interestingly, the relative importance of cytolytic properties of CD8^+^ T cells has been questioned by *in vivo* CD8^+^ depletion experiments in SIV-infected nonhuman primates [21, 22]. The lack of a prolonged lifetime of infected CD4^+^ T cells in the absence of CD8^+^ lymphocytes suggested that non cytolytic responses might represent another strong element of the protective CD8^+^ T cell-mediated response to HIV or SIV infection. The presence of CD8^+^ T cell mediated noncytotoxic antiviral response (CNAR) in HIV-infected individuals has been reported more than two decades ago [23–26]. This activity has also been demonstrated in CD8^+^ cells from simian immunodeficiency virus (SIV)mac infected macaques and baboons, SIVsm-infected sooty mangabeys, SIVagm-infected African green monkeys, HIV-1-infected chimpanzees, in feline immunodeficiency virus infection and an EBV-specific cell line [27–34]. This non-cytolytic activity can be detected *in vitro* as early as one week after infection and thus appears to be a part of innate antiviral immune response of CD8^+^ cells [29, 34]. Whereas the originally proposed factor termed CD8 T cell secreted Antiviral Factors (CAF) has not been identified yet, numerous studies identified other soluble host factors inhibiting viral replication [35, 36]. These secreted factors include several beta chemokines as well as RNases (Angiogenin, RNase 4) and potentially exosomes [17, 37–41]. Activated primary human T cells can also produce soluble factors that mediate downregulation of CD4 in macrophages which render them refractory to HIV-infection. [42].

All these evidences point towards a strong involvement of CNAR as an important innate immune modulator. However, its direct correlation with disease progression in LTNPs has not been systematically studied yet. To assess the impact of CNAR on disease progression, we investigated longitudinally a cohort of SIV-infected LTNPs which also included monkeys that lost CNAR during the observation period. Results show that the CNAR represents a stable characteristic in the majority of aviremic LTNPs and is correlated inversely with plasma viral load. Furthermore, CNAR responsive CD4^+^ T cells are lost when viral load increases.

## Materials and Methods

### Animals

SIVmac239 and SIVmac251-infected rhesus macaques (*Macaca mulatta*) of Indian origin were recruited from several experiments conducted at the German Primate Centre (‘Deutsches Primatenzentrum’, DPZ). Major histocompatibility complex (MHC) class I genotyping was carried out as described before [43].

### Ethical statement

All relevant protocols strictly adhere to the German Animal Welfare Act which follows the European Union guidelines on the use of non-human primates for biomedical research. All experiments were approved by an external ethics committee authorized by the Lower Saxony State Office for Consumer Protection and Food Safety (project license AZ 33.9-42502-04-12/0820), issued by the same State Office.

All macaques were housed and treated at the DPZ under permanent surveillance by veterinarians and animal caretakers. Procedures for animal welfare and to ameliorate suffering were undertaken in accordance with the recommendations of the Weatherall report “The use of nonhuman primates in research”. The monkeys were kept with a 12:12 light-dark schedule at a temperature between 18–23°C and a humidity range of 50–60%. All nonhuman primate rooms are provided with 8–10 fresh air changes per hour. The macaques had constant water access and were fed twice a day with dry food supplemented with fresh fruits. Environmental enrichment was provided by placing rings and perches into the cages, by foraging or task-oriented feeding methods (e.g. treats, vegetables or fruits frozen in ice cubes, food puzzle), and by playing music. Blood samples were drawn from monkeys sedated with 10 mg ketamine i.m. per kg body weight. In cases of mild suffering predefined by a scoring system of termination criteria that was approved by the external ethics committee and corresponds to the IACUC endpoint guidelines, monkeys were euthanized. The scoring system considers independently weight loss, anomalies in defecation, water and food consumption, behaviour (attention/alertness, potential movement disorders), respiration, size of lymph nodes, and the hemogram. Animals were humanely euthanized by an overdose of Pentobarbital-Natrium (Narcoren^®^, Merial, Hallbergmoos, Germany) under anesthesia.

### Viral RNA quantification

Viral RNA was isolated from frozen plasma samples or cell culture supernatant either following the MagAttract Virus Mini M48 protocol (Qiagen, Hilden, Germany) or, manually, employing QIAamp Viral RNA Mini Kit (Qiagen, Hilden, Germany). Purified SIV RNA was quantified using TaqMan-based real-time PCR (Quantitect probe RT-PCR Kit; Qiagen, Hilden, Germany) on an ABI-Prism 7500 sequence detection system (Applied Biosystems) as described by supplier using gag forward (5´-ACCCAGTACAACAAATAGGTGGTAACT-3´), gag reverse primer (5´-TCAATTTTACCCAGGCATTTAATGT-3´) and a fluorescent labeled probe (5´-6FAM(6-carboxyfluorescein)-TGTCCACCTGCCATTAAGCCCGAG-TAMRA(6-carboxytetramethylrhodamine-3´)[44]. The detection limit for viral RNA was between 35–67 copies per ml plasma depending on the extraction method.

### Viral inhibition assay

CD4^+^ cells and CD8^+^ cells were isolated by magnetically labelled antibodies cell separation technique (MACS) for nonhuman primates (Miltenyi Biotech, Bergisch Gladbach, Germany). For routine viral inhibition assay, CD4^+^ cells from SIV-uninfected, MHC-mismatched donors and CD8^+^ from experimental animals were used. In some experiments the flow-through after isolation of CD8^+^ lymphocytes was used for infection as indicated. Purified CD4^+^ and CD8^+^ cells were re-suspended at a density of 2 × 10^6^ cells/ml in RPMI complete medium [RPMI 1640 (PAN Biotech, Aidenbach, Germany) supplemented with 10% fetal calf serum (PAN Biotech), 100 U/ml penicillin (PAN Biotech) and 100 μg/ml streptomycin (PAN Biotech)], and activated with Concanavalin-A (ConA) (10 μg/ml; SERVA Electrophoresis GmbH, Germany) for 24 hours respectively. Cells were washed and CD4^+^ cells were infected with SIVmac239 at a multiplicity of infection (MOI) 0.001 TCID_50_ for 90 minutes with intermittent swirling every 15 min. After washing thrice with complete RPMI medium cells were re-suspended at a concentration of 1 × 10^6^ cells/ml in the RPMI complete medium supplemented with 100 U/ml recombinant human IL-2 (PeproTech, Hamburg, Germany). *In vitro* infected CD4^+^ T cells were either cultured alone (controls) or co-cultured with ConA-activated CD8^+^ cells from experimental animals in duplicate or triplicate depending on the cell yield at a 1:2 cell input ratio (5 × 10^5^ CD4^+^ T cells: 1 × 10^6^ CD8^+^ T cells) in each well of a 24 flat bottom well plate (Becton Dickinson GmbH, Heidelberg, Germany). Supernatants were collected at day 5 and 7 for viral RNA isolation. CD8^+^ cells collected from one experimental animal were routinely tested with SIV-infected CD4^+^ cells from two or three different donor monkeys. Fold inhibition was calculated as geometric mean of viral RNA copies per ml supernatant from the control divided by geometric mean of viral RNA copies per ml supernatant of respective co-cultures. For assessing CNAR the median from the two or three assays with different CD4^+^ cells performed at one time point was calculated.

To assess autologous viral replication CD4^+^ T cell were purified as described above, stimulated with ConA for 24 h. Viral RNA copies in cell culture supernatant were determined 7 days later. In two animals that presented with high autologous viral replication (M2201, M12672) viral inhibition test was performed without adding exogenous virus. In most experiments CD4+ and CD8+ cells were isolated from frozen lymphocytes.

### Definition of CNAR

We had to introduce a quantitative definition for CNAR since we assessed viral load by quantitative RealTime PCR, which is different compared to HIV- viral inhibition assays (e.g. reverse transcriptase measurements [20]). For SIV-infected LTNPs we used as estimate ≥50fold inhibition to distinguish CNAR from any kind of background activity of CD8 cells. The same cut-off was used for uninfected macaques except that one macaque with a mean of 48fold inhibition was also regarded as CNAR positive.

### Transwell cultures

For transwell cultures, 1 x 10^6^ ConA-activated, SIV-infected CD4+ T cells isolated by MACS from uninfected donors or the flow-through after CD8 cell isolation from SIV-infected monkeys were resuspended in 1.5 ml complete RPMI 1640 and placed into the lower well of 6 well transwell plate (Thermo Fisher Scientific Inc., Nunc polycarbonate membrane inserts in Multidish 6). 1 x 10^6^ CD8^+^ cells isolated by MACS were re-suspended in 500μl complete RPMI and placed into the transwell insert. Viral load was measured at day 7 post infection. Control cultures without CD8^+^ cells contained 1 x10^6^ cells re-suspended in 1 ml complete RPMI 1640 supplemented with IL-2.

### Flow cytometric analysis

T cell subpopulations were analyzed by polychromatic flow cytometry. 0.5–1 x 10^6^ PBMCs or 50 μL of whole blood were stained for 30 min at room temperature in the dark with the following antibodies: anti-CD3 Alexa Fluor700 (clone SP34-2), anti-CD4 HorizonV450 (clone L200), anti-CD28 PerCP-Cy5.5 (clone L293), anti-CD95 FITC (clone DX2), anti-CD197 (CCR7) PE-Cy7 (clone 3D12), HLA-DR APC-Cy7 (clone L243) from BD Biosciences (Heidelberg, Germany), anti-CD27 Brilliant Violet 650 (clone O323) and anti-PD-1 (CD279) APC (clone EH12.2H7) from BioLegend (San Diego, USA), anti-CD8 Pacific Orange (clone 3B5) (Invitrogen, Thermo Fisher Scientific Inc., USA) and anti-CD45RA ECD (clone 2H4) from Beckman Coulter (Krefeld, Germany). Lysis of residual RBCs and fixation was performed using RBC lysis/fixation solution (BioLegend, San Diego, USA) for 15min at room temperature in the dark, followed by an additional washing step.

For intranuclear staining of Ki67 the FOXP3 Fix/Perm buffer set (BioLegend, San Diego, USA) was used according to the manufacturer’s instructions. In brief, following surface staining with anti-CD4 APC-Cy7 (clone OKT4), anti-CD8 APC (clone SK1) (BioLegend), and Live/dead fixable Violet Dead cell stain (Invitrogen, Thermo Fisher Scientific) for 30min, cells were incubated with Fix/Perm solution and subsequently with Perm buffer. Cells were then stained with anti-Ki67 PerCP-Cy5.5 (clone B56, BD Biosciences) diluted in Perm buffer for 30 min, followed by an additional washing step.

For analysis of necrotic cells, cells were surface stained with anti-CD4 APC-Cy7 (clone OKT4), anti-CD8 APC (clone SK1) (BioLegend), and Live/dead fixable Violet Dead cell stain for 30min, washed twice with PBS/BSA, and fixed in 0.3% formaldehyde solution. Cells were acquired using a BD LSRII cytometer with FACS DIVA software 6.1.3. T cell populations were analyzed using FlowJo 9.6 (TreeStar). Flow cytometry gating strategy for T cells is shown in S3 Fig.

### Statistical analysis

For statistical analyses GraphPad Prism version five or Sigma Plot 10 was employed. P values <0.05 were considered as significant. Tests for determining significance are indicated together with the results. Mann Whitney *U* test was used assess whether two groups differ significantly from each other, spearman rank correlation was employed to test whether there exists a statistical dependence of two variable parameters, e.g. whether CNAR correlates significantly with viral RNA copies. Kruskal Wallis test and Dunn's test for multiple comparisons were used assess significant differences between several groups.

## Results

### Aviremic LTNPs display durable CD8^+^ mediated noncytotoxic viral inhibition

The ability of CD8^+^ T cells to suppress viral replication *in vitro* was investigated in 21 SIV-infected long term non-progressors (LTNPs) over a period of up to 6 years, 16 uninfected and 10 SIV-infected rhesus macaques with progressing disease mainly by cross-sectional studies. Two of the uninfected macaques became LTNPs after experimental SIV-infection (total number of LTNPs = 23). LTNPs were defined as monkeys either surviving SIV-infection for more than 1 year and having plasma viral RNA copies below the detection limit of our assay or surviving SIV-infection more than 3 years post infection. At time of initial testing, LTNPs had survived SIV-infection for 1 to 6 years and had plasma viral RNA copies below 5000/ml (S1 and S2 Tables). The observation period after the final CNAR test in most cases exceeded one year in order to verify the LTNP-status at time of testing (S1 Table). Progressors were defined as SIV-infected macaques with plasma viral RNA copies >10,000 per ml, and/or CD4 cell counts <200/μl. In this group, three macaques were included that had lost the LTNP status (S3 Table).

An acute infection assay similar to as described by Mackewicz and colleagues was employed to assess CNAR [45]. In order to avoid inhibition of viral replication mediated by CTL-responses, the CD4^+^ and CD8^+^ T cells used in the viral inhibition test were at least mismatched in their well-expressed *MHC* class I alleles and/or in *MHC* class I alleles that could contribute to SIV-specific CTL-responses [43, 46, 47].

Considering that the antiviral activity of the CD8^+^ cells also depended on the CD4^+^ T cells, it was attempted to keep the number of donor macaques to a minimum. Therefore, CD4^+^ cells from three donors were used to test CD8^+^ cells from the majority of LTNPs and uninfected macaques at least once. Albeit viral replication in CD4^+^ cells varied, no systematic influence of viral replication pattern in CD4^+^ cells on CNAR was observed (S1 Fig). In addition we found no correlation between number of Ki67^+^ and necrotic CD8^+^ cells at onset of co-cultures and at the day 3 co-culture and CNAR (S2 Fig). At day 3, Ki67^+^ CD8^+^ cells had increased but again no correlation between abundance of these cells and CNAR was evident (S2B Fig).

**Fig 1:**
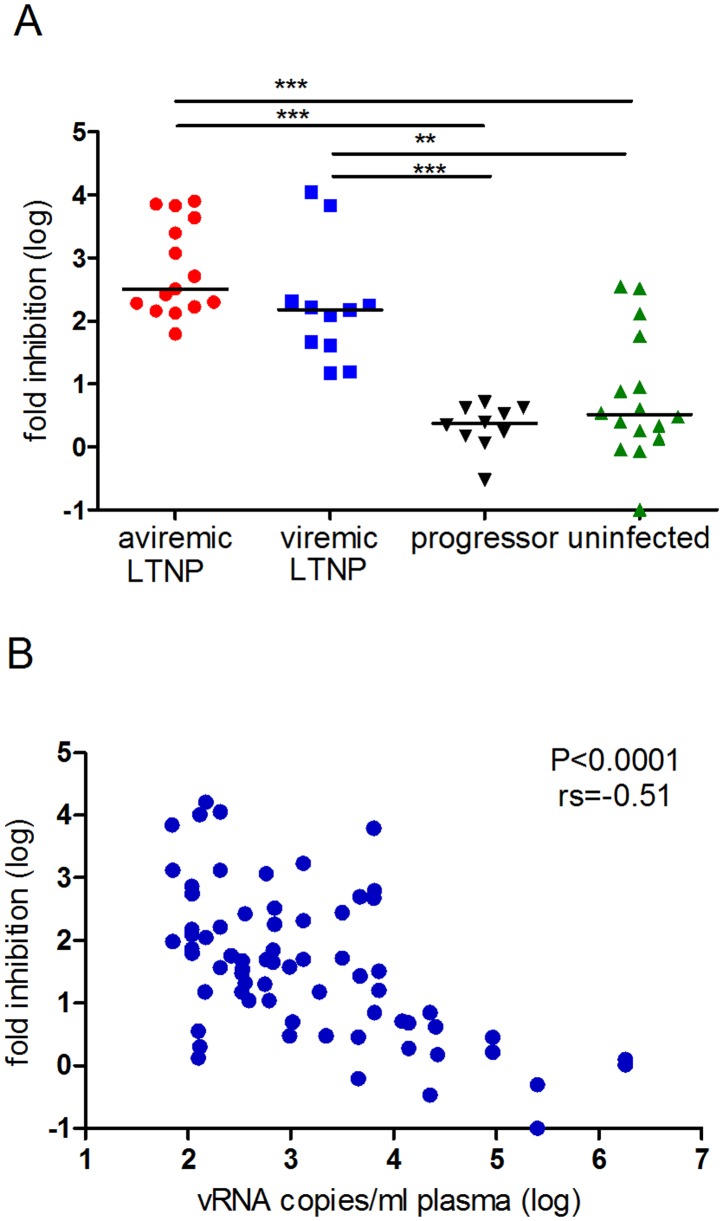
CNAR is strongest in LTNPs and correlates inversely with viral load. (A) CNAR was significantly stronger in SIV-infected LTNPs compared to uninfected macaques and progressors. Viral inhibition was tested with CD8^+^ cells from 15 aviremic and 11 viremic (geometric mean: 1780 viral RNA copies/ml plasma) long-term non-progressing macaques (LTNPs), 16 uninfected macaques and 10 SIV-infected macaques with progressing disease (geometric mean: 2.41E+04 viral RNA copies/ml plasma). Each dot represents the median of up to 10 viral inhibition tests performed with CD8^+^ cells from one macaque (mean number of tests per animal: 3.3, STABW = 2.4). Significance (nonparametric Kruskal Wallis test followed by Dunn's test for multiple comparison) is indicated by asterisks (*: p<0.05, **: p<0.01, ***: p<0.001). Median is indicated. (B) CNAR correlated inversely with plasma viral load. Fold viral inhibition (log) was assessed in 70 assays, each performed in triplicate, with CD8^+^ cells from in total 11 viremic LTNPs and 10 progressors (mean number of assays per animal = 3.3, STABW = 2.8) and correlated significantly with plasma viral load (spearman rank order correlation, p<0.0001, rs = -0.51). Data were taken from S2 and S3 Tables.

**Fig 2:**
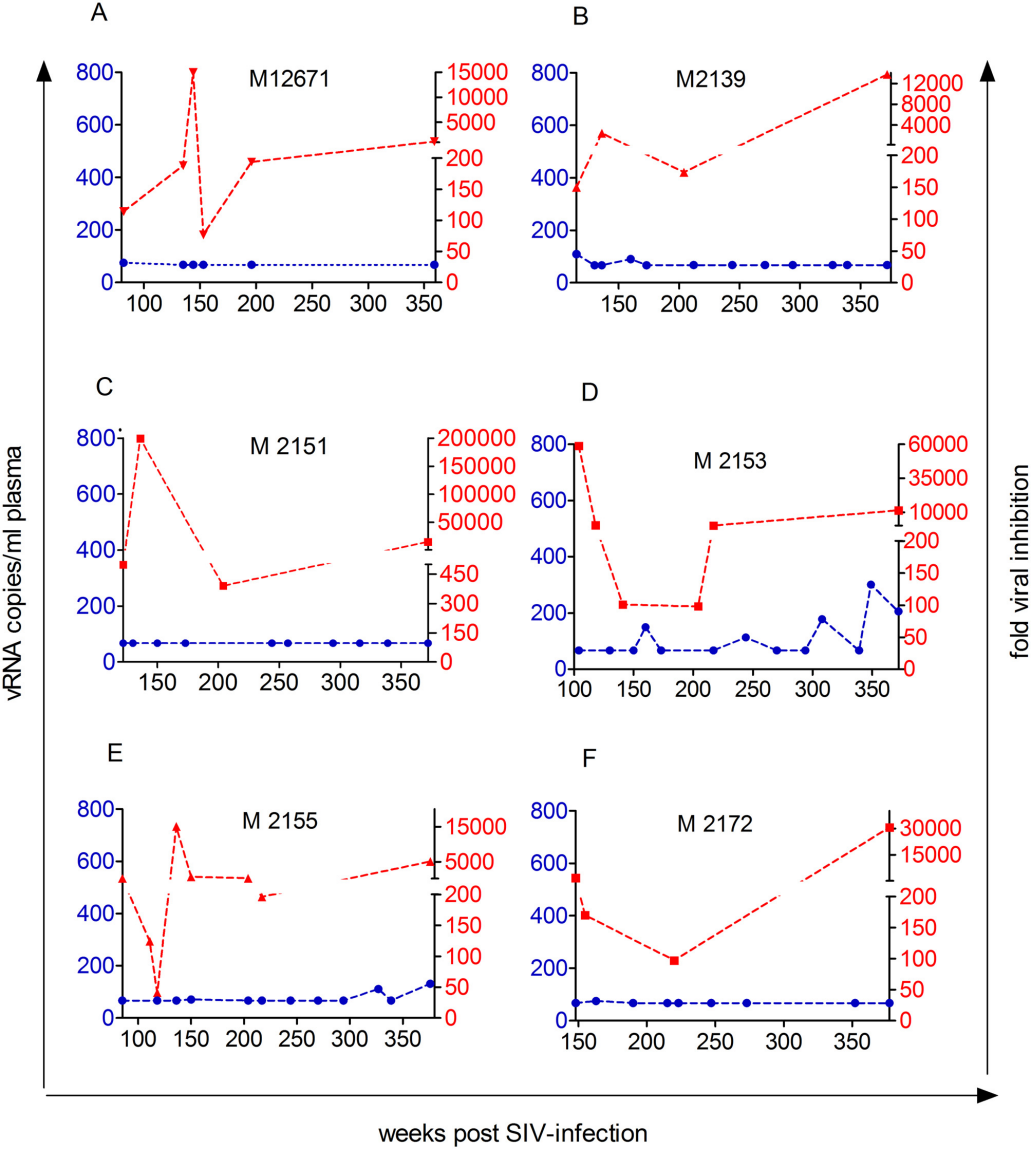
Longitudinal analysis of *in vitro* viral inhibition and plasma viral RNA copies in SIV-infected LTNPs with undetectable or low plasma viral load. CD8^+^ cell-mediated inhibition of viral replication (red dashed line) and viral RNA copies per ml plasma (blue dashed line) was measured over a period of up to 6 years. Data are shown from the first time point when viral inhibition test was performed, the time line depicts weeks post SIV-infection (wpi). One point typically reflects the mean inhibition performed with SIV-infected CD4^+^ cells from two different donors. Respective monkey IDs are shown in each graph.

**Fig 3:**
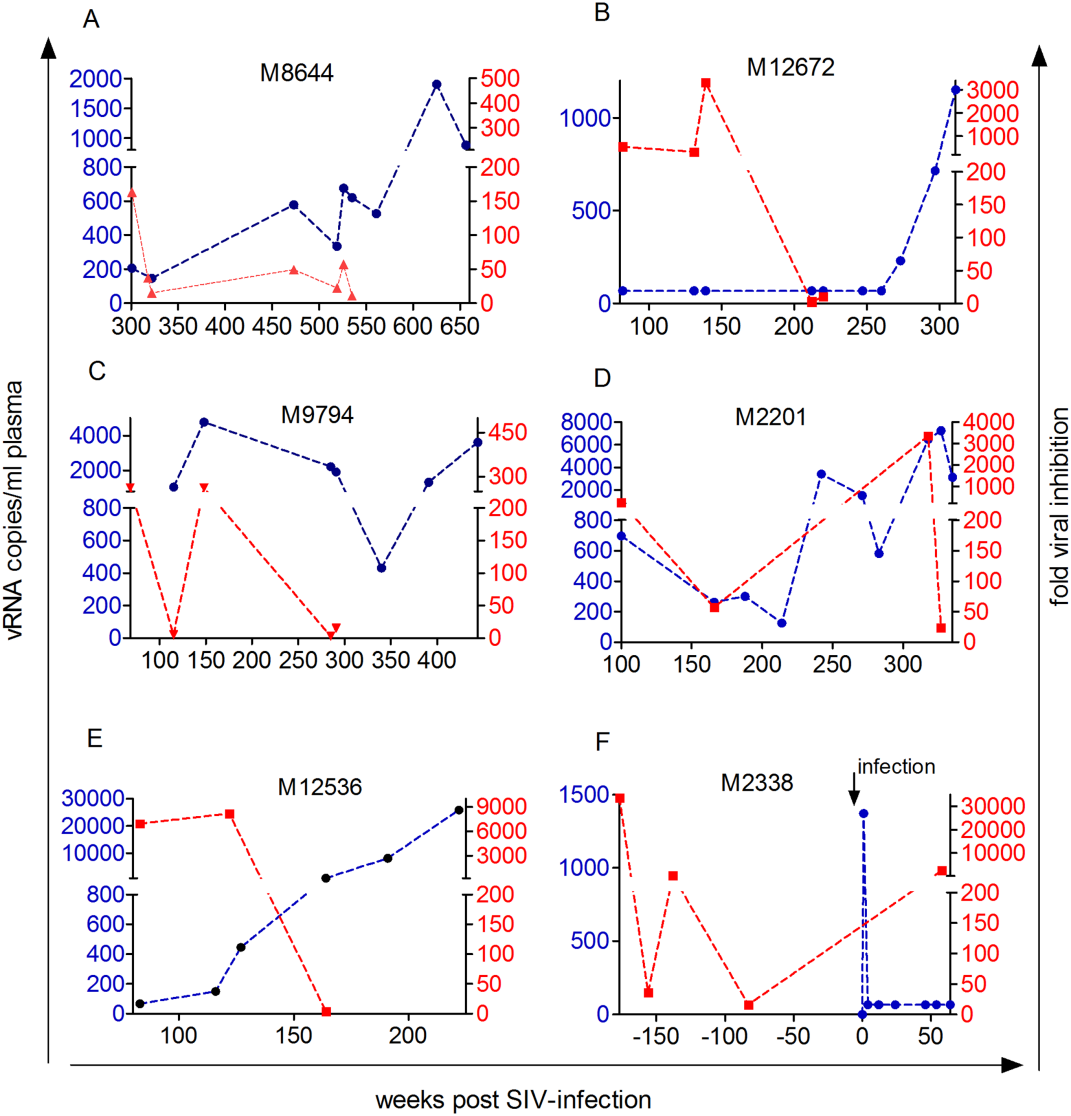
Longitudinal analysis of CD8-mediated *in vitro* viral inhibition (red dashed line) and plasma viral load. (blue dashed line) in five LTNPs that finally lost CNAR (A-E), and one macaque pre and post infection (F). Time line starts when first viral inhibition test was performed. One time point typically reflects the mean of fold inhibition determined with CD4^+^ cells from two donors. Respective monkey IDs are shown in each graph.

CD8^+^ cells from 19 of 23 LTNPs displayed CNAR and inhibited *in vitro* viral replication 50 to 60.000fold at seventh day post *in vitro* infection (S2 Table). Aviremic LTNPs (elite controller) displayed higher CNAR than viremic LTNPs (median: 324 vs. 150fold inhibition, p = 0.05, Mann Whitney *U* test, Fig 1A). Note, that one viremic LTNP displaying high CNAR showed only sporadic low level of virus replication (M2153, Fig 2D). Both viremic as well as aviremic LTNPs displayed significant CNAR as compared to uninfected macaques and progressors (p<0.002; significant for multiple testing, Kruskal Wallis and Dunn's multiple comparison test, Fig 1A). Moreover, CNAR correlated inversely with plasma viral load (spearman rank correlation: p<0.0001, r = -0.51, Fig 1B). Long-term measurements were performed in 11 macaques that had plasma viral RNA copy numbers around the detection limit at the start of study (Figs 2A–2F and 3A–3E). In most aviremic animals CNAR was a stable characteristic of CD8^+^ cells over years albeit the extent of the activity could vary by 2 to 3 logs (Fig 2A–2F). Only one macaque suppressed viral replication below the detection limit for seven years, but displayed CNAR (fold inhibition ≥50) only in two out of five assays performed at three different time points (M2284).

### Temporal association between loss of CNAR and increase of plasma viral load

CNAR was assessed at multiple time points in five LTNPs where the activity was lost after a period of time (Fig 3A–3E). One macaque (Fig 3A) remained asymptomatic now for 13 years albeit it was never able to suppress plasma viral load below the detection limit of our assay for longer periods of time, and thus displayed a very rare phenotype. The other four lost CNAR (Fig 3B–3E), displayed increasing viral load and three progressed to AIDS (Fig 3B, 3C and 3E). One macaque lost CNAR about one year before an increase in viral load (Fig 3B), whereas CNAR was still observed at the initial phase of the increase of viral load in three macaques but was lost after a further increase (Fig 3C–3E).

In summary, CNAR appeared to be a stable characteristic of CD8^+^ cells in the vast majority of aviremic LTNPs. It was ultimately lost when viral load continued to increase but no tight temporal relationship between loss of CNAR and rising viral load was observed.

### Detection of CNAR in uninfected macaques

We assessed the presence of a potential pre-existing CNAR in CD8^+^ cells from 16 uninfected animals. CD8^+^ cells from four monkeys were able to inhibit viral replication 16 to 30,000fold, although the extent of activity was in uninfected macaques on average, significantly lower as lower compared to SIV-infected LTNPs (median: 3fold vs. >150fold, Fig 1A). Five of the uninfected macaques were half-sibs. They were derived from a breeding group where nearly all 11 descendants that had a favourable *MHC* class I genotype (Mamu-A1*001 and Mamu-B*017) became LTNPs after SIV-infection. These *Mhc* alleles are associated with slow disease progression, but do not exclusively explain the LTNP status. Notably, CD8^+^ cells from three out of five of these sibs displayed CNAR while only one out of 11 macaques derived from other breeding groups showed strong CNAR. This observation hints at the possible involvement of some genetic factors in displaying CNAR, as well as a potential contribution of CNAR to the LTNP status.

In one uninfected monkey of the above mentioned breeding group, CNAR was assessed four times over a period of 96 weeks (Fig 3F, S3 Table). The CD8^+^ cells were able to inhibit viral replication at least 16fold at all time points indicating that CNAR itself may represent a stable feature of CD8^+^ cells, at least in relatively stable conditions as provided in an experimental setting. As expected, this animal also displayed CNAR after infection and controlled plasma viral load to undetectable levels (Fig 3F).

### Presence of CNAR indicates functional, anti-SIV-specific CD8 T cell compartment

In the classical viral inhibition assay heterologous, MHC mismatched CD4^+^ cells are used to assess the presence of CNAR which, however, does not inform about the anti-virus specific activity of CD8^+^ cells in autologous cells. Therefore, we investigated CD8^+^ cell-mediated viral inhibition in autologous CD4^+^ T cells.

**Fig 4:**
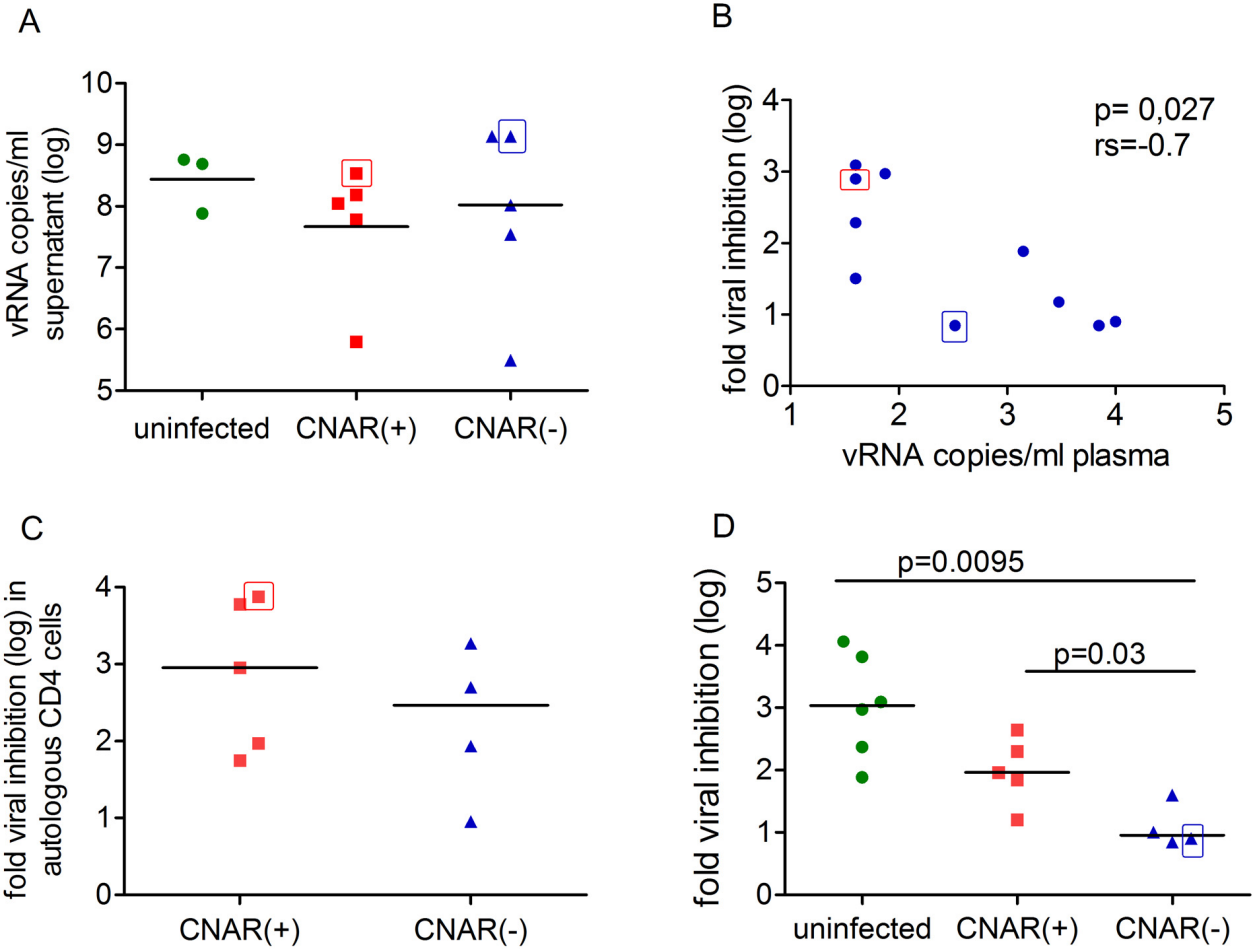
CNAR depends also on origin of CD4+ cells. (A) Viral RNA copies (log) at day 7 after *in vitro* infection of purified CD4^+^ cells or CD8^+^ depleted cells from five CNAR(+) macaques (M2172, M13907, M13909, M13923-A, M13929, Table 1), five CNAR(-) (M2284, M13929, M2201, M13919, M12672, Table 2) and three uninfected macaques (donor 1–3). Median is shown by a horizontal line. Each point represents the geometric mean of at least three infection experiments with cells from one animal. The three groups do not differ significantly. M13929 was first tested positive (indicated by red edging, Table 1) and later negative for CNAR (indicated by blue edging, Table 2). (B) Fold inhibition of viral replication assessed with CD8^+^ cells from five CNAR(+) and five CNAR(-) LTNPs co-cultured with SIV-infected CD4^+^ cells from uninfected macaques (donor 1 and/or donor 2) correlated with plasma viral RNA copies (p = 0.027, rs = -0.7). Animal IDs are same as in (A). (C) Fold inhibition (log) of viral replication in SIV-infected CD4^+^ cells co-cultured with autologous CD8^+^ cells from five CNAR(+) (M2172, M13907, M13909, M13923-A, M13929, Table 1) and four CNAR(-) (M2284, M2201, M13919, M12672, Table 2) LTNPs. Median is indicated by a horizontal line. CD8^+^ cells from CNAR(+) inhibited SIV replication in autologous CD4^+^ cells on average stronger compared to CNAR(-) monkeys but the difference is not significant (median: 902 vs. 293 fold; p = 0.41, Mann Whitney *U* test). (D) CNAR depends on the origin of CD4^+^ cells. Fold viral inhibition (log) assessed with CD8^+^ cells from CNAR positive monkeys (M2172, M13907 and M13909) and *MHC* mismatched SIV-infected CD4^+^ cells from uninfected monkeys, CNAR(+) monkeys (M2172, M13907, M13909, M13923-B) and viremic CNAR(-) LTNPs (M13929, M2201, M13919, M12672). Inhibition of viral replication is significantly lower in *in vitro* SIV-infected CD4^+^ T cells from viremic CNAR(-) LTNPs compared to CD4^+^ T cells from uninfected macaques and CD4^+^ T cells from CNAR positive LTNPs after co-culture with CNAR(+) CD8^+^ cells. P values are indicated (Mann Whitney *U* test). Data are taken from Tables 1 and 2. Median is shown by a horizontal line. Data point with CD4+ cells from CNAR(-) M13929 is edged by blue lines.

Cultures with CD4^+^ cells from SIV-uninfected monkeys were included for comparative reasons. After *in vitro* infection, CD4^+^ cells from CNAR(+) and CNAR(-) LTNPs and uninfected macaques replicated SIV to a similar extent (Fig 4A). Viral inhibition tests with CD4^+^ cells from uninfected donor 1 and 2 confirmed the inverse correlation of CNAR with plasma viral load (p = 0.027, rs = -0.7, Fig 4B). CNAR(+) macaques (Table 1, fold inhibition in bold) were on average more than threefold better able to limit viral replication in autologous CD4^+^ cells than CNAR negative macaques (Table 2, fold inhibition shown in bold) albeit the difference was not significant (median: 902 vs. 293fold inhibition; Fig 4C). Presence of CNAR thus indicates that CD8^+^ cells can suppress viral replication also well in autologous CD4^+^ cells by cytolytic and potentially non cytolytic means, whereas absence of CNAR can indicate impairment of CD8^+^ function.

**Table 1:** Fold viral inhibition mediated byCD8^+^cells from CNAR(+) SIV-infected macaques.

**Animal ID: CD8^+^ cells**	**M2172** ^a^	**M2172**	**M13929**	**M13923**	**M13923**	**M13907**	**M13909**
*In vivo* infecting SIVmac	239	239	251	251	251	251	251
vRNA copies/ml plasma	≤40^b^	≤40^b^	≤40^b^	≤40^b^	≤40^b^	75	1410
		Fold inhibition					
**Assay ID**	A,C	B		A	B		
**Animal ID: CD4** ^**+**^ **cells**							
Uninfected Donor 1	6540	1236			10	940	77
TW-donor 1	4				1	5	6
Uninfected Donor 2		234	790	2216	325		
TW-donor 2		0.17	1000	10			
Uninfected Donor 3		11540	22	2			
TW-donor 3		0.5	2	2			
SIV-infected M2172	**1467**	**337**			16	200	440
TW-M2172	**3**	**2**					
SIV-infected M13929			**6000**	5000^c^			
TW-13929			**1**	36			
SIV-infected M13923		12	16000^c^	**7500**	**306**		
TW-13923			126	**9**			
SIV-infected M13907	92					**94**	
SIV-infected M13909	70					3	**56**
TW13909	1						

^a^No carrier of Mamu-A*01, -B*17, -B*08; *MHC*-mismatched with cells from other SIV-infected monkeys

^b^ detection limit

^c^ co-culture with Mamu-A*01 matched macaque

TW = transwell. Numbers in bold: Test with autologous cells.

**Table 2:** Fold viral inhibition mediated by CD8^+^ cells from CNAR(-) SIV-infected macaques.

**Animal ID: CD8** ^**+**^ **cells**	**M2172** ^a^	**M2284**	**M13929**	**M2201** ^c^	**M13919**	**M12672** ^a^ ^,^ ^c^
*In vivo* infecting SIVmac:	239	251	251	251	251	239
vRNA copies/ml plasma	40^b^	40^b^	330	3000	7000	10000
vRNA copies/ ml supernatant cultured CD4^+^ cells	≤40^b^	≤40^b^	800	3,11E+05		3,45E+07
		Fold inhibition				
**Animal ID: CD4** ^**+**^ **cells**						
Uninfected Donor 1	3660–18750	32	7	15	7	8
SIV-infected M2284	8094	**1853**			817^d^	
SIV-infected M13929	7					
SIV-infected M2201	10			**86**		
SIV-infnected M13919	8	12^d^			**500**	
SIV-infected M12672	40			16		**9**

^a^ No carrier of Mamu-A*01, -B*17, -B*08; *MHC*-mismatched with cells from other SIV-infected monkeys

^b^ detection limit

^c^ Test with autologous CD4^+^ cells without adding exogenous virus

^d^ co-culture with Mamu-A*01 matched macaque

Bold: Test with autologous cells.

### CNAR depends upon the functionality of both, CD4^+^ and CD8^+^ T cells

Loss of viral restriction by non cytolytic mechanisms can be explained by the loss of CNAR-expressing CD8^+^ cells as measured in the classical viral inhibition assay [45], the emergence of viral escape mutants [48], an increase in CD4^+^ cells non-responsive to CNAR, or potentially even by increasing concentration of soluble host proteins fostering viral replication. To get further insight into the biological meaning of CNAR we applied cross-wise viral inhibition tests with CD4^+^ and CD8^+^ cells from SIV-infected monkeys (Tables 1 and 2), and investigated whether the biological impact of CNAR depends also on origin and functionality of the CD4^+^ T cells. All available LTNPs except of two (M2172: Fig 2F, M12672: Fig 3D) carried Mamu-A1*001 alone or in combination with another *MHC* class I allele known to be associated with elite control. To avoid strong cytolytic activity attributed to the presence of Mamu-A1*001 or Mamu-B*017, the majority of the experiments were performed with cells from CNAR positive, Mamu-A1*001 and Mamu-B*017 negative M2172 (S1 Table). Transwell assays were performed to investigate whether CNAR was mediated by soluble factors (Table 1).

CNAR was mainly mediated by cell-cell-contact which is in line with previous results [25, 26]. For instance, CD8^+^ cells from M2172 and M13923 displayed strong viral inhibitory activity in autologous (>1000fold) and heterologous cultures, but low to no inhibitory effect in most transwell cultures (≤10fold). However, CD8^+^ cells from M13929 mediated some, but highly variable, level of viral inhibition by soluble factors in transwell assays depending on the origin of CD4^+^ T cells (0–1000 fold) indicating that CNAR can be multifactorial in nature exemplified by the requirement for physical contact (Table 1).

Furthermore, inhibition of viral replication was significantly lower in CD4^+^ T cells from viremic CNAR(-) macaques compared to CD4^+^ T cells from uninfected macaques or CNAR positive macaques (Fig 4D). Moreover, CD8^+^ cells from CNAR(+) M2172 were able to inhibit viral replication in autologous CD4^+^ cells, in cells from the CNAR(-) elite controller M2284, two CNAR(+) monkeys (M13907, 13909), and in CD4^+^ T cells from 8 uninfected donors assessed in long-term measurements, but its CD8^+^ cells could not inhibit viral replication in CD4^+^ cells from five monkeys with increasing viral load that had lost or were about losing CNAR (M2201, M12672, M13919, M13929, M13923-B; Tables 1 and 2). Similarly, viral replication in CD4^+^ cells from CNAR^+^ M2172 was inhibited by CD8^+^ cells from CNAR(+) monkeys (M13909 and M13907, Table 1).

Finally, the results demonstrate that CNAR and the responsiveness of CD4^+^ cells towards it are two independent properties. For example, CD8^+^ cells of the elite controller M2284 showed weak CNAR (determined at three time points), but its CD4^+^ cells responded well to CNAR (>8000fold, Table 2). On the opposite, CD8^+^ cells of M13907 and M13909 showed CNAR (mean 415fold inhibition), but viral replication in their CD4^+^ cells was less well inhibited by CD8^+^ cells from CNAR-positive M2172 (mean 66fold) or autologous CD8^+^ cells (mean 75fold, Table 1).

### T cell subsets in CNAR(+) and CNAR(-) LTNPs

**Fig 5:**
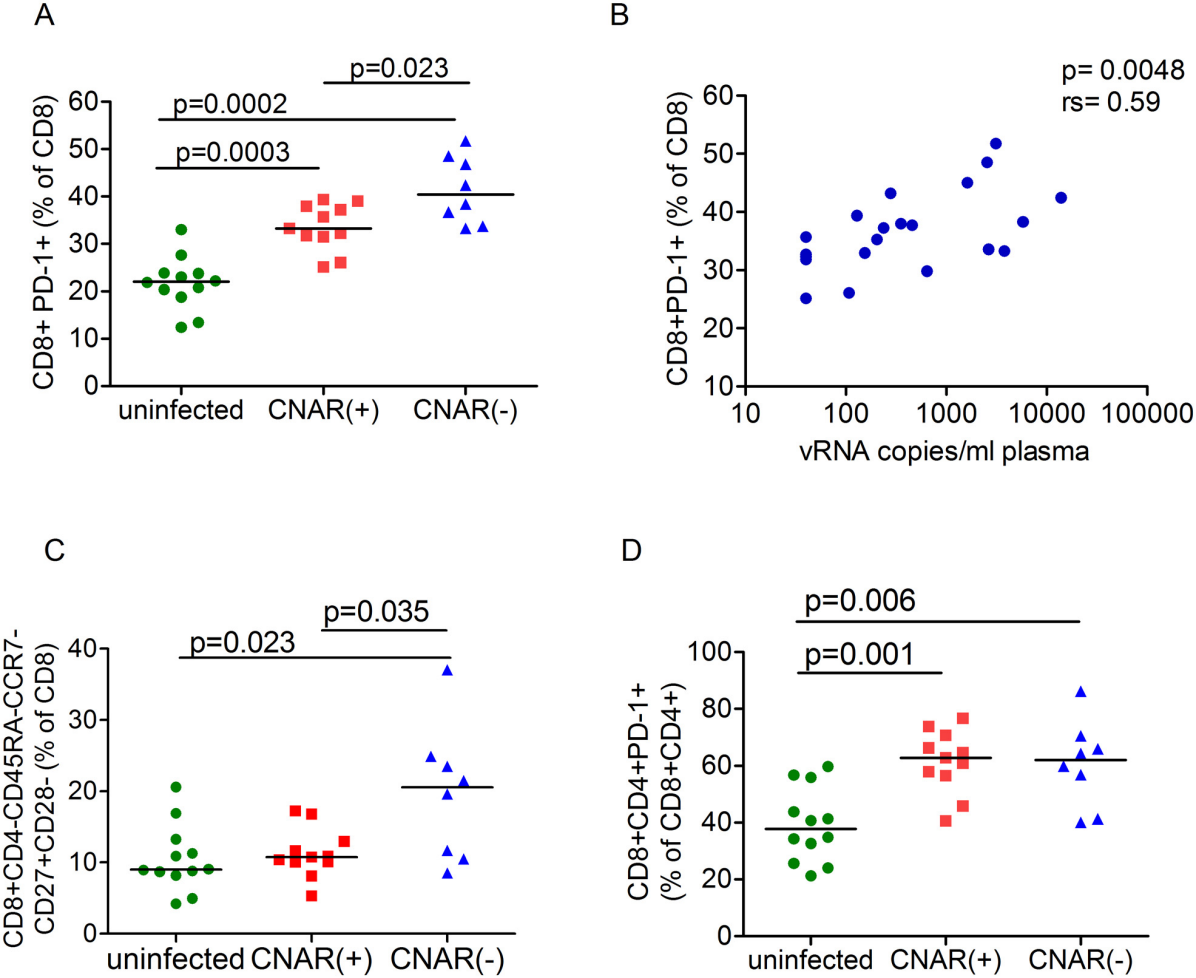
Frequencies of T cell populations in SIV-infected CNAR(+), CNAR(-) and uninfected macaques. (A) Frequency of CD8^+^PD-1^+^ cells (percentage of CD3^+^CD8^+^) are significantly higher in CNAR(-) LTNPs compared to CNAR(+) LTNPs and uninfected monkeys. P values (Mann Whitney *U* test) are shown. (B) Frequency of CD8^+^PD-1^+^ cells (percentage of CD3^+^CD8^+^) correlate with plasma SIV RNA copies in LTNPs (n = 21). Data represent the mean from three independent measurements. P value and spearman r are indicated. (C) Frequency of transitional memory cells (CD3^+^CD8^+^CD4^−^CD45RA^−^CCR7^−^CD27^+^CD28^−^; percentage of CD3^+^CD8^+^) are significantly higher in CNAR(-) LTNPs compared to CNAR(+) or uninfected macaques. (D) Frequencies of CD4^+^CD8^+^PD-1^+^ cells (percentage of CD3^+^CD4^+^CD8^+^) are significantly higher in SIV-infected LTNPs [CNAR(+) or CNAR(-)] compared to uninfected macaques. Results from uninfected macaques represent the mean from two independent measurements of each animal (n = 12). Data in Fig 5A, 5C and 5D from CNAR(+) (n = 11) and CNAR(-) (n = 8) macaques represent the mean of up to 3 time points, each two months apart. P values (Mann Whitney *U* test) are shown. Horizontal lines depict the median.

Since it has been reported that CNAR may be mediated by transitional memory cells and to some extent by PD-1 expressing CD8^+^ T cells [20], we investigated the relative frequencies of different T cell subsets in SIV-infected CNAR positive and negative as well as in uninfected macaques by flow cytometry. Data from three time points each two months apart was analyzed. CNAR was tested at least once within this time period in animals with stable plasma viral load. In animals with increasing viral load CNAR was assessed again or FACS data were excluded for analysis. Mean frequencies of naïve (CD28^+^ CD95^−^), CD4^+^ or CD8^+^ central memory (T_CM_, CD28^high^ CD95^+^) and effector memory (T_EM_, CD28^low/-^ CD95^+^) cells did not differ between CNAR positive or negative monkeys. However the frequency of PD-1^+^ CD8^+^ cells differed significantly between the groups (Fig 5A; S3 Fig). Similar to humans, PD-1^+^CD8^+^ cells were lowest in uninfected individuals (Fig 5A)[20]. Furthermore, PD-1^+^CD8^+^ (given as frequency of PD-1^+^ of CD8^+^) correlated with plasma viral load (rs = 0.59, p = 0.0048, Fig 5B). Since in SIV-infected macaques CNAR was strongest in aviremic LTNPs, we found no evidence that PD-1 expressing CD8^+^ cells contribute to viral inhibition (Fig 5B).

A similar picture emerged for transitional memory (CD45RA-CCR7-CD27+CD28-) CD8+ cells. Percentages of transitional CD8^+^ T cells were lowest in uninfected individuals, marginally higher in CNAR(+) individuals and significantly highest in CNAR(-) individuals (Fig 5C).

Double positive (CD8^+^CD4^+^) T cells can constitute a considerable fraction of T cells in macaques ranging from 1 to 20% of CD3^+^ cells in this study. In HIV-infected individuals the frequency of double positive (DP) cells was found to be increased in advanced disease [49]. However, neither the percentage of CD4^+^CD8^+^ cells (of CD3^+^ cells), nor the percentage of transitional memory (CD3^+^CD8^+^CD4^+^CD45RA^−^CCR7^−^CD27^+^CD28^−^) among CD4^+^CD8^+^ cells varied significantly between uninfected, CNAR(+) or CNAR(-) macaques (p>0.14, data not shown). PD-1^+^ cells (of CD4+CD8+) were significantly higher in CNAR(+) and CNAR(-) macaques compared to uninfected individuals (p≤0.006, Mann Whitney test), but did not differ between CNAR(+) and CNAR(-) macaques (Fig 5D). Taken together, similar to HIV-infected humans percentages of transitional memory cells and PD-1^+^ CD8^+^ T cell were increased in SIV-infected macaques compared to uninfected individuals, but in contrast to humans, these T cell subsets were not increased in CNAR(+) macaques.

## Discussion

The presence of soluble factors suppressing HIV or SIV replication secreted by CD8^+^ and CD4^+^ cells has been reported repeatedly by cross-sectional studies [36, 39, 50]. We report here a longitudinal measurement of CNAR in a cohort of LTNPs including five that finally lost control of viral replication. We used an acute infection assay that, in addition to the activity of soluble factors, allowed the interaction between CD8^+^ and CD4^+^ T cells either through cell-cell contacts or through the secretion of exosomes [41]. Since the CD4^+^ T cells were mismatched for *MHC* class I alleles, viral inhibition due to cytolytic activity can be largely excluded [6, 20, 23]. Moreover such type of assay may be physiologically more relevant as compared to transwell assay [51–53].

In this study, CD4^+^ and CD8^+^ lymphocytes were stimulated with ConA. Since the study was initiated at a time when ConA stimulation was commonly used, we kept the procedure for comparative reasons. However, presence of CNAR has also been demonstrated in unstimulated CD8 cells [20, 54].

A strong long-term CNAR was detected over a time period of up to 6 years in the majority of aviremic LTNPs. Furthermore, CNAR correlated inversely with plasma viral load similar as described earlier [29, 45, 55–58].

CD8^+^ T cells that can mediate CNAR have not been unequivocally identified. Furthermore, research in SIV-infected macaques is largely lacking in this respect. In addition, it is likely that there are fundamental differences between CNAR mediated by soluble substances or cell-cell contact explaining differential results which should be an issue for future studies. Transitional memory cells have been reported as the main fraction of CD8^+^ cells and PD-1^+^CD8^+^ as minor fraction that secrete HIV inhibitory substances [20]. Our findings do not support a strong role for transitional memory cells and PD-1^+^ cells for CNAR in the macaque model of AIDS. The discrepancy to the above mentioned results may be due to the fact that we detected CNAR mainly in aviremic LNTPs with physiological frequencies of transitional memory cells, whereas in humans CNAR was mainly detected in viremic LTNPs which had—like viremic macaques -higher levels of transitional memory and PD-1^+^ CD8^+^ cells.

Since, CD8^+^ cells from SIV-infected CNAR-positive macaques were also able to control viral replication in autologous CD4^+^ cells presence of CNAR indicates an intact CD8^+^ cell compartment. However, CNAR and SIV-specific cytolytic activity can represent two distinct activities among CD8^+^ T cells. For example, we did not observe a tight temporal association between loss of CNAR and increasing viral load in all macaques. Two macaques that had lost CNAR were still able to inhibit viral replication in autologous cells probably by their cytolytic activity. Albeit it is conceivable that also antigen-specific, probably nef- and gag-specific T cells, contribute to non cytolytic viral suppression [40, 52], our findings as well as those from other groups describing CNAR also in CD8^+^ cells from uninfected humans and monkeys [29, 31, 33] indicate that CNAR is not uniquely displayed by SIV-specific CD8^+^ cells. A report on discordance between the secretion of cytokines and cytolytic function of CD8^+^ cells may point into the same direction [59]. Moreover, studies in humans have shown that CNAR expressing CD8^+^ cells have a biased T cell receptor repertoire [20] indicating that CNAR may be displayed by an unknown specialized subset of CD8^+^ T cells that need not be necessarily SIV-specific T cells.

Notably, we observed large variations in CNAR (Tables 1 and 2, S2 and S3 Tables). However, variations were lowest in CNAR(-) progressors. Viral replication pattern in CD4^+^ cells was not a significant factor to explain the variations. In addition, no correlation between number of Ki67^+^ and necrotic CD8^+^ cells at onset of co-cultures and CNAR was detected. However, an influence of varying numbers of apoptotic or necrotic CD8^+^ cells or the quality of CD4^+^ T cells which may be not adequately reflected by the ability to replicate SIV on CNAR can have influenced the variations found especially in CNAR(+) macaques.

Moreover, the variability may arise from the intrinsic individual differences in the reactivity of CD4^+^ cells, from fluctuations in CNAR-mediating CD8^+^ cells and responsive CD4^+^ cells, and potentially a yet not identified polymorphic factor. Cell-contact mediated CNAR may depend on some polymorphic host factor since the extent of viral inhibition depended on both, the origin of CD4^+^ and CD8^+^ cells. An involvement of host factors that can mediate suppression of SIV-replication in a cell-cell contact dependent mechanism and do not represent a classical HLA class I or class II gene has been postulated earlier [60]. This report shows that CNAR depended also on the functionality of CD4^+^ cells. The responsiveness of CD4^+^ cells to CNAR was significantly lower in viremic CNAR negative macaques compared to CD4+ cells from macaques with undetectable viral load or uninfected macaques. Our results imply that not only CD8^+^ cells can lose CNAR but (for the first time) that also CD4^+^ T cells can lose their ability to respond to CNAR when viral load increases but retain the ability to support viral replication. It has been reported that CD4^+^ T cells from HIV-patients on effective therapy have a reduced ability to respond to CTL-mediated lysis compared to CD4^+^ cells from elite controllers [61]. The differential susceptibility of CD4^+^ cells can shape viral reservoir and contribute to long-term survival. It remains to be investigated whether differential susceptibility of CD4^+^ cells to CNAR can also contribute to long-term survival.

The quest to identify CD8 T cell antiviral factors remains a promising field in the search of natural antiviral substances, and will add to the functional profile of protective immune responses required for the design and interpretation of AIDS vaccine experiments. Future studies may reveal whether and how some (specialized) CD8^+^ T cells provide a stimulus for CD4^+^ T cells to undergo a status transformation that does not support viral replication, and /or induce secretion of antiviral factors by CD4^+^ cells themselves [50, 62, 63]. An important step towards this goal will be the identification of receptor-ligand pairs on CD4^+^ and CD8^+^ cells that are involved in cell-contact mediated CNAR. Further investigation will reveal whether an improved understanding of this activity can be translated into therapeutic interventions. HIV positive blood cancer patients, for example, can be treated by bone marrow transplantation [64, 65]. While allogeneic bone marrow transplantation from CCR5^+^ donors has not ultimately lead to eradication of HIV in all recipients [66], it may be, for instance, worthwhile to test whether the selection of donors whose CD8^+^ T cells are able to limit viral replication by CNAR in the recipient can add to the anti-HIV treatment.

## Conclusions

The current longitudinal study in the macaque model of AIDS identifies CNAR as a stable element of the immune response in SIV-infected LTNPs that can be detected in some uninfected macaques as well. CNAR was strongest in aviremic LTNPs corroborating that CNAR is a component of an immune system with an excellent capability to suppress viral replication. Further tests confirmed that presence of CNAR is also associated with suppression of viral replication in autologous CD4^+^ cells, whereas absence of CNAR can indicate impairment of CD8^+^ function. CNAR expressing CD8^+^ cells and responding CD4^+^ cells are presented as two separable properties that were lost with increasing viral load. Loss of CNAR can mark a transition from an LTNP-status to a progressing infection, but does not immediately lead to immunodeficiency in viremic controllers. In the macaque model of AIDS, physical contact between CD8^+^ and CD4^+^ cells appears to be the most important aspect for mediating CNAR and viral inhibition, which depends upon the origin of both, CD8^+^ and CD4^+^ cells. Moreover, CNAR is multifactorial in nature as demonstrated by the differential requirement for physical contact between CD8+ and CD4+ cells. A strong role of transitional CD8^+^ T cells in supporting CNAR in the macaque model of AIDS could be excluded. Further insight into molecular mechanisms and especially an improved knowledge about CD8^+^ and CD4^+^ cells involved in this phenomenon could pave ways for novel immune-based therapies for HIV infections.

## Supporting Information

S1 Fig No significant influence of viral replication in CD4^+^ cells on CNAR. Viral RNA copies in supernatant of CD4^+^ control cells is compared with fold viral inhibition assessed after incubation with CD8^+^ cells from (A) LTNPs, (B) progressors, (C) uninfected macaques at day 7 post initiation of co-cultures. (D-F) depict the results obtained with CD4^+^ cells from three most often used donors (2163, 2249, 2232) co-cultivated with CD8^+^ cells from LTNPs, and (G) fold viral inhibition assessed over time in CNAR(+) macaque (M2172) as example. Open squares show results obtained with CD4 cells from monkey 2232, closed circles with CD4 cells from other donors.
(PDF)

S2 Fig No correlation between percentages of Ki67^+^ and necrotic CD8^+^ cells and CNAR at start of co-cultures. (A) Percentages of necrotic CD8^+^ cells (of total CD8^+^ cells) were analyzed by flow cytometry in co-cultures with *in vitro* SIV-infected CD4^+^ cells from one uninfected donor (2232, Donor 1) and CD8^+^ cells from 3 viremic (M2219, M13907, M13929) (vRNA copies/ml plasma: 145, 525, 545) and 3 aviremic LTNPs (M2172, M13913, M13923). One aviremic LTNP had switched to CNAR(-) status (M13913, indicated by gray edging), and two were CNAR(+). Lymphocytes were processed for FACS-analysis immediately after addition of CD8^+^ cells to SIV-infected CD4^+^ cells (day 0) and at day 3 after initiation of the co-cultures. For the cultures, lymphocytes initially frozen in liquid nitrogen were used. Data were related to fold inhibition of viral replication obtained in assays performed in parallel. (B) Percentages of Ki67^+^ CD8^+^ cells (of live CD8^+^ cells) at day 0 and day 3 post initiation of the cultures described in (A) and fold inhibition of viral replication (CNAR) is depicted. No correlation between percentages of Ki67^+^, necrotic CD8^+^ cells and CNAR was found (p>0.6 spearman rank correlation). (C) Percentage of necrotic CD8^+^ cells from three uninfected macaques processed identically as the aviremic and viremic LTNPs. One of them (gray edging) was found to be CNAR(-), the others were not tested.
(PDF)

S3 Fig Flow cytometry gating strategy for CD8^+^ and CD4^+^ CD8^+^ DP transitional memory cells as well as CD8^+^ and CD4^+^ CD8^+^ DP PD-1^+^ cells. Representative gating of T cell subsets in whole blood is depicted. Excision of duplets (a singlet gate) was followed by gating on lymphocytes and subsequent gating on CD3^+^ T cells. T cells were further divided into CD4^+^, CD8^+^ and CD4^+^ CD8^+^ DP cells. CD8^+^ and CD4^+^ CD8^+^ DP transitional memory cells were identified by gating on CD197^−^ CD45RA^−^ CD28^−^ CD27^+^ cells.
(PDF)

S1 Table Overview on long-term non progressing SIV infected macaques: survival (total), survival post final CNAR test and follow up, *MHC* class I alleles. (XLSX)

S2 Table Data for Figs 1, 2 and 3: Fold inhibition mediated by CD8^+^ cells of long-term non progressing SIV infected macaques with undetectable and detectable viral load and viral RNA copies /ml plasma. (XLSX)

S3 Table Data for Figs 1, 2 and 3: Fold inhibition mediated by CD8^+^ cells of naïve macaques and SIV-infected progressing macaques with viral RNA copies /ml plasma. (XLSX)
