# Supplementary figures and images for: Noncytolytic CD8+ Cell Mediated Antiviral Response Represents a Strong Element in the Immune Response of Simian Immunodeficiency Virus-Infected Long-Term Non-Progressing Rhesus Macaques
Source: PLoS One. 2015 Nov 9;10(11):e0142086. doi: 10.1371/journal.pone.0142086 (PMC4638345; doi:10.1371/journal.pone.0142086)

**Additional Figure S1**

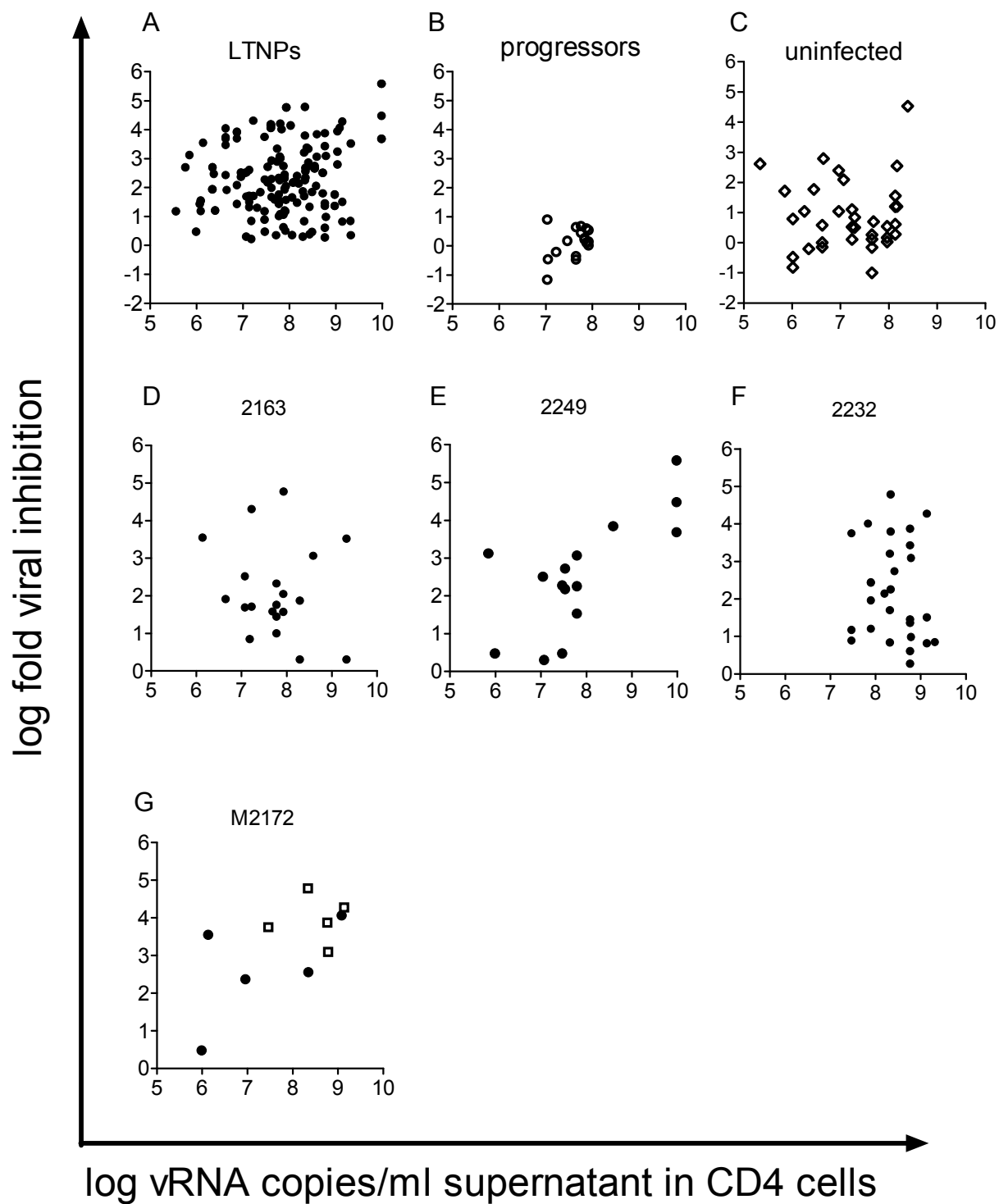

Supplement: S1 Fig — Viral RNA copies in supernatant of CD4+ control cells is compared with fold viral inhibition assessed after incubation with CD8+ cells from (A) LTNPs, (B) progressors, (C) uninfected macaques at day 7 post initiation of co-cultures. (D-F) depict the results obtained with CD4+ cells from three most often used donors (2163, 2249, 2232) co-cultivated with CD8+ cells from LTNPs, and (G) fold viral inhibition assessed over time in CNAR(+) macaque (M2172) as example. Open squares show results obtained with CD4 cells from monkey 2232, closed circles with CD4 cells from other donors. (PDF) [file pone.0142086.s001.pdf]

Figure S2

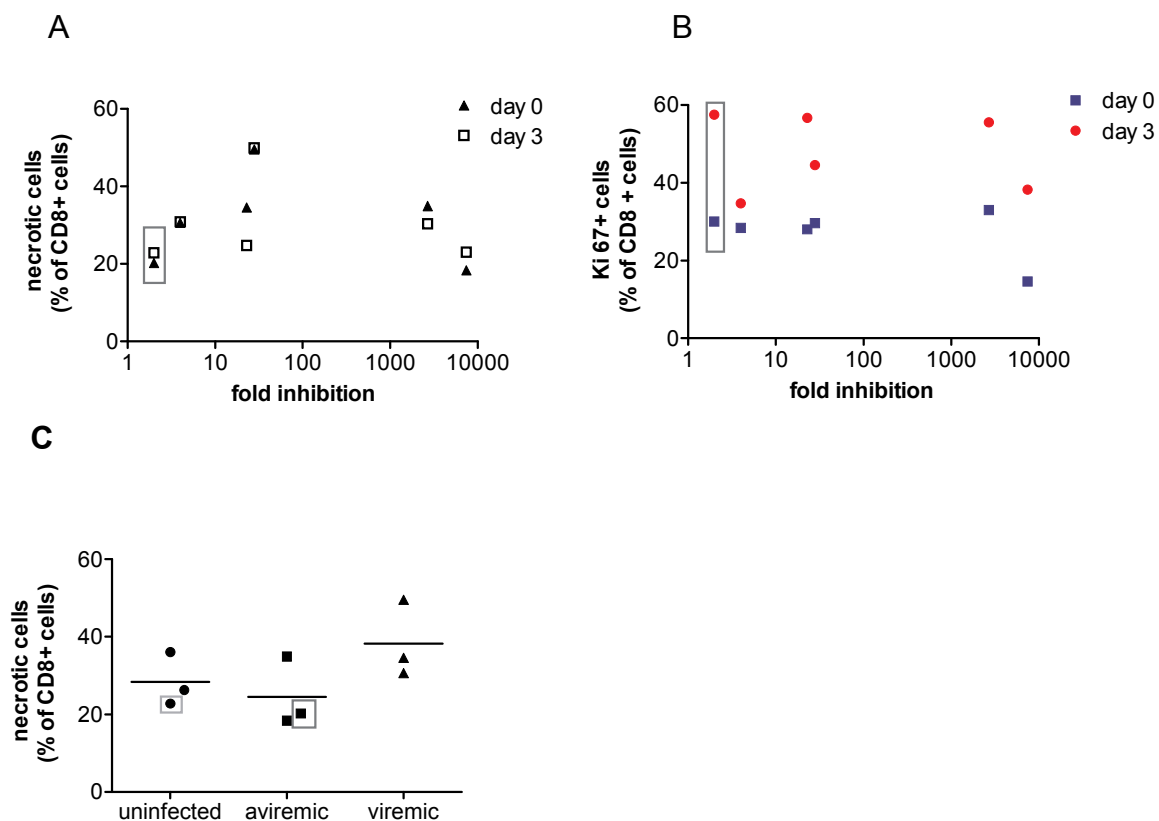

Supplement: S2 Fig — (A) Percentages of necrotic CD8+ cells (of total CD8+ cells) were analyzed by flow cytometry in co-cultures with in vitro SIV-infected CD4+ cells from one uninfected donor (2232, Donor 1) and CD8+ cells from 3 viremic (M2219, M13907, M13929) (vRNA copies/ml plasma: 145, 525, 545) and 3 aviremic LTNPs (M2172, M13913, M13923). One aviremic LTNP had switched to CNAR(-) status (M13913, indicated by gray edging), and two were CNAR(+). Lymphocytes were processed for FACS-analysis immediately after addition of CD8+ cells to SIV-infected CD4+ cells (day 0) and at day 3 after initiation of the co-cultures. For the cultures, lymphocytes initially frozen in liquid nitrogen were used. Data were related to fold inhibition of viral replication obtained in assays performed in parallel. (B) Percentages of Ki67+ CD8+ cells (of live CD8+ cells) at day 0 and day 3 post initiation of the cultures described in (A) and fold inhibition of viral replication (CNAR) is depicted. No correlation between percentages of Ki67+, necrotic CD8+ cells and CNAR was found (p>0.6 spearman rank correlation). (C) Percentage of necrotic CD8+ cells from three uninfected macaques processed identically as the aviremic and viremic LTNPs. One of them (gray edging) was found to be CNAR(-), the others were not tested. (PDF) [file pone.0142086.s002.pdf]
