# Supplementary material for: Noncytolytic CD8+ Cell Mediated Antiviral Response Represents a Strong Element in the Immune Response of Simian Immunodeficiency Virus-Infected Long-Term Non-Progressing Rhesus Macaques
Source: PLoS One. 2015 Nov 9;10(11):e0142086. doi: 10.1371/journal.pone.0142086 (PMC4638345; doi:10.1371/journal.pone.0142086)

Supplementary Figure

S2. Flow cytometry gating strategy for CD8^{+} and CD4^{+} CD8^{+} DP transitional memory cells as well as CD8^{+} and CD4^{+} CD8^{+} DP PD-1^{+} cells. Representative gating of T cell subsets in whole blood. Excision of duplets (a singlet gate) was followed by gating on lymphocytes and subsequent gating on CD3^{+} T cells. T cells were further divided into CD4^{+}, CD8^{+} and CD4^{+} CD8^{+} DP cells. CD8^{+} and CD4^{+} CD8^{+} DP transitional memory cells were identified by gating on CD197^{-} CD45RA^{-} CD28^{-} CD27^{+} cells.

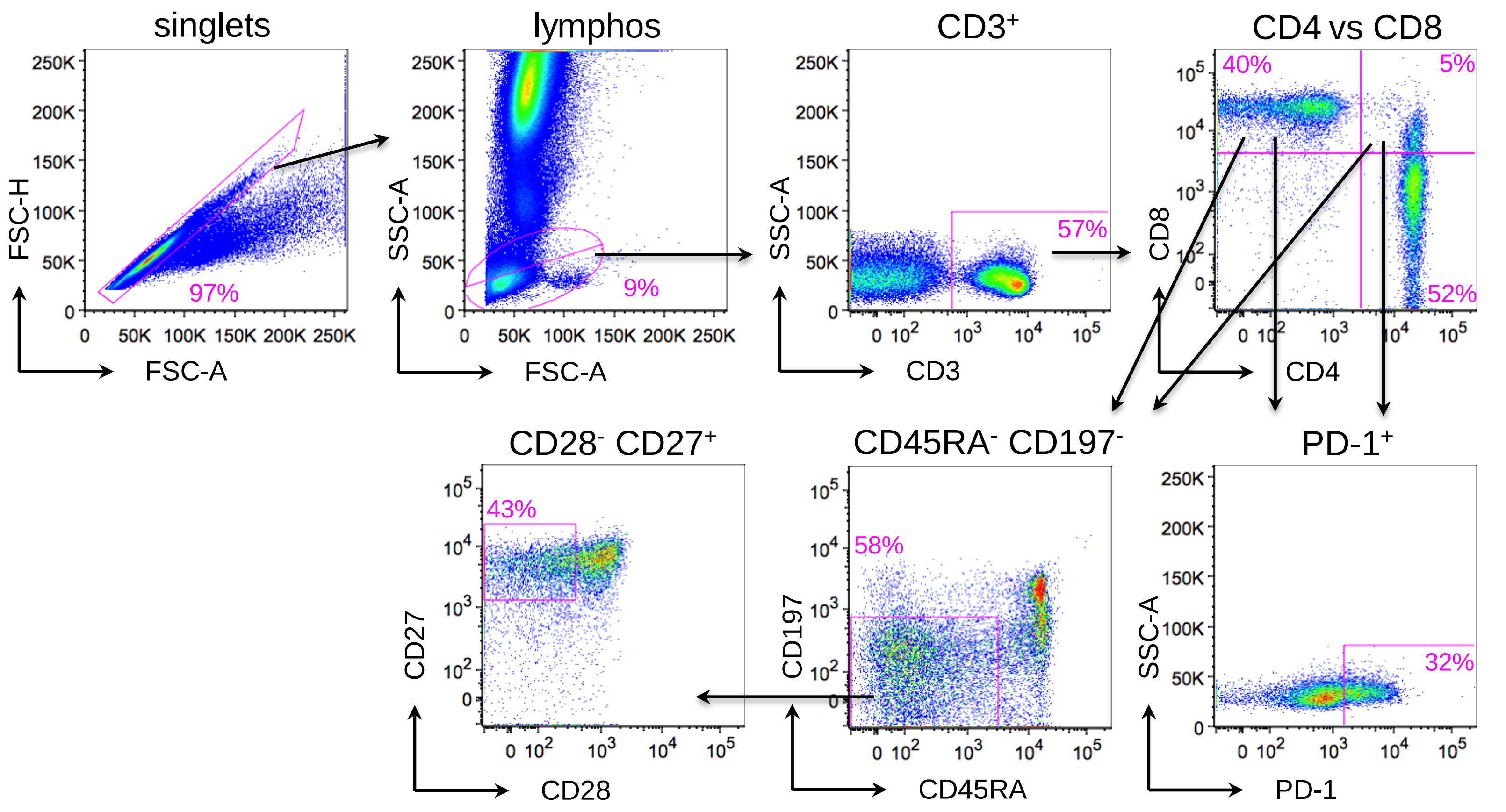

Supplement: S3 Fig — Representative gating of T cell subsets in whole blood is depicted. Excision of duplets (a singlet gate) was followed by gating on lymphocytes and subsequent gating on CD3+ T cells. T cells were further divided into CD4+, CD8+ and CD4+ CD8+ DP cells. CD8+ and CD4+ CD8+ DP transitional memory cells were identified by gating on CD197− CD45RA− CD28− CD27+ cells. (PDF) [file pone.0142086.s003.pdf]
